# Similarities and dissimilarities between psychiatric cluster disorders

**DOI:** 10.1038/s41380-021-01030-3

**Published:** 2021-01-27

**Authors:** Marissa A. Smail, Xiaojun Wu, Nicholas D. Henkel, Hunter M. Eby, James P. Herman, Robert E. McCullumsmith, Rammohan Shukla

**Affiliations:** 1 Department of Pharmacology and Systems Physiology, University of Cincinnati, Cincinnati, OH USA; 2 Neuroscience Graduate Program, University of Cincinnati, Cincinnati, OH USA; 3 Department of Neurosciences, University of Toledo College of Medicine and Life Sciences, Toledo, OH USA; 4 Veterans Affairs Medical Center, Cincinnati, OH USA; 5 Department of Neurology, University of Cincinnati, Cincinnati, OH USA; 6 Neurosciences Institute, ProMedica, Toledo, OH USA

**Keywords:** Psychiatric disorders, Neuroscience, Psychology

## Abstract

The common molecular mechanisms underlying psychiatric disorders are not well understood. Prior attempts to assess the pathological mechanisms responsible for psychiatric disorders have been limited by biased selection of comparable disorders, datasets/cohort availability, and challenges with data normalization. Here, using DisGeNET, a gene-disease associations database, we sought to expand such investigations in terms of number and types of diseases. In a top-down manner, we analyzed an unbiased cluster of 36 psychiatric disorders and comorbid conditions at biological pathway, cell-type, drug-target, and chromosome levels and deployed density index, a novel metric to quantify similarities (close to 1) and dissimilarities (close to 0) between these disorders at each level. At pathway level, we show that cognition and neurotransmission drive the similarity and are involved across all disorders, whereas immune-system and signal-response coupling (cell surface receptors, signal transduction, gene expression, and metabolic process) drives the dissimilarity and are involved with specific disorders. The analysis at the drug-target level supports the involvement of neurotransmission-related changes across these disorders. At cell-type level, dendrite-targeting interneurons, across all layers, are most involved. Finally, by matching the clustering pattern at each level of analysis, we showed that the similarity between the disorders is influenced most at the chromosomal level and to some extent at the cellular level. Together, these findings provide first insights into distinct cellular and molecular pathologies, druggable mechanisms associated with several psychiatric disorders and comorbid conditions and demonstrate that similarities between these disorders originate at the chromosome level and disperse in a bottom-up manner at cellular and pathway levels.

## Introduction

Psychiatry encompasses a vast number of disorders and comorbidities. While these disorders have their own unique traits, common molecular mechanisms may be involved in their underlying pathology [1]. Identifying such common elements would enhance our understanding of numerous disorders simultaneously and identify common therapeutics against them.

Prior efforts to identify such common mechanisms were informative yet incomplete. Most studies [2, 3], in an attempt to make the data more manageable, compared the transcriptomic profiles of a few diseases at a time, limiting their ability to reveal patterns across a wide range of conditions and comorbidity. The diseases included in these studies were selected based on disease severity, known associations, or data/cohort availability [4, 5], thus preventing the exploration of novel relationships (e.g., depression and epilepsy [6]). Furthermore, normalization often presents a challenge in these studies [7, 8], and efforts to unionize diverse datasets can compromise results and limit the conclusions drawn from them.

**Fig. 1:**
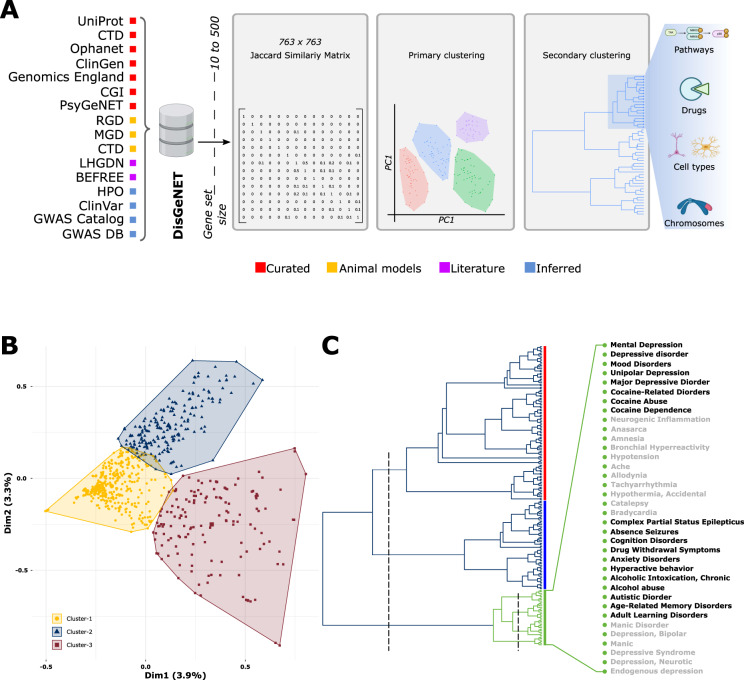
Disease-associated gene-sets fall into three distinct clusters. **A** Workflow used to derive the “psychiatric cluster” in an unbiased top-down manner. Curated gene-sets form different sources cataloged in DisGeNET’s were filtered by gene-set size, compared via a Jaccard similarity matrix and sorted into global clusters of similar disease using principal component analysis. Then, a primary cluster enriched in psychiatric, immune, metabolic, and neurodegenerative disorders was further broken down based on gene-set similarity, ultimately yielding the “psychiatric cluster” of 36 diseases that was used for subsequent analysis of pathways, drugs, cell-types, and chromosomes. **B** A PCA based clustering of 763 curated disorders. Note that cluster 1 falls at the center of the plot indicating a low internal correlation between the disorders in this cluster. **C** Hierarchical clustering of Cluster 2 disorders. An initial cutting of three (left dashed line) reveals three branches shown in red, blue, and green. The green branch (aka the psychiatric cluster) was considered for all subsequent analysis. Further cutting the green branch (right dashed line) reveals four subgroups composed of 36 disorders labeled on the right. See Supplementary Fig. 1 for further details. CTD Comparative Toxicogenomics Database, ClinGen The Clinical Genome Resource, CGI The Cancer Genome Interpreter, PsyGeNET Psychiatric disorders Gene association NETwork, RGD Rat Genome Database, MGD Mouse Genome Database, LHGDN Literature-derived Human Gene-Disease Network, HPO Human Phenotype Ontology, GWAS Gene-Wide Association Study.

Another approach to this comprehensive analysis is to draw on publicly available gene-disease databases. These resources have greatly expanded in number and detail in the past several years; thus, enhancing the simultaneous comparison of several diseases. One such resource is DisGeNET [9, 10], a knowledge management platform cataloging genes associated with human diseases. Utilizing diverse resources, comprised of human, animal, and computational data, DisGeNET identifies gene-disease associations (GDAs) and curates signature gene-lists associated with each disease (Fig. 1A). Presently, DisGeNET catalogs disease-associated gene-sets for 24,166 diseases, featuring 628,685 GDAs across 17,549 genes [9]. As these gene-lists are not reliant upon expression profile, it precludes the limitations that have compromised prior efforts to compare psychiatric disorders. Furthermore, the sheer number of GDAs makes this an ideal platform to compare numerous related and diverse diseases in an unbiased manner.

Here, we use curated, evidence-supported and clinically relevant disease-associated gene-sets from DisGeNET to identify unbiased clusters of similar diseases; then, with special emphasis on a cluster enriched with psychiatric disorders and associated comorbidities, we performed a series of in silico analyses to understand the biological complexity of these disorders at pathways, cell-types, drug-targets, and chromosomes levels. To distill the information and compare the disorders at each level, we propose a novel metric called density index. Improving the understanding of the psychiatric disease landscape, this top-down approach is critical to identifying highly related disorders in an unbiased manner, revealing common and unique mechanisms underlying disease pathology and pointing to the biological level of complexity which drives the similarities and dissimilarities between disorders.

## Methods

Disease-specific curated gene-lists were downloaded from DisGeNET (https://www.disgenet.org/downloads). Detailed methods for finding disease–disease similarity, filtering for psychiatric cluster disorders, gene ontology (GO) analysis, density index, cell-type and drug-target enrichment, chromosome overrepresentation, and Rand index (RI) are available in the online supplements.

## Results

### Disease profiles fall into three distinct clusters

To look for molecularly similar diseases, we calculated a pairwise similarity matrix for 763 disease-associated gene-sets from DisGeNET (Fig. 1A). A principal component analysis over this matrix segregated the disease profiles into three distinct clusters (Fig. 1B; Supplementary Table 1). Positioned at the center, Cluster 1 showed fluidic boundaries with the other clusters and contained 422 profiles. Most diseases in this cluster were congenital, syndrome, familial, sex-linked, and autosomal in origin, but few psychiatric disorders (including schizophrenia) and cancer were also observed. A majority of disease in this cluster fell below the (0, 0) coordinates, suggesting uncorrelated disease of low overlapping gene-sets (Supplementary Table 1). Cluster 2 and Cluster 3 were orthogonal to each other and each contained highly correlated diseases. Cluster 2 contained 192 profiles and was primarily composed of diseases related to psychiatric disorders, inflammation, metabolism, and neurodegeneration. Cluster 3 contained 149 profiles and was primarily composed of various types of cancer.

Overall, the separation of disease profiles into three clusters suggests that converging mechanisms are involved in the presentation of diseases within each cluster. Given our group’s focus on psychiatric disorders, the remainder of this paper will focus specifically on Cluster 2.

### Hierarchical clustering reveals a distinct subgroup of psychiatric disorders

Hierarchical clustering of Cluster 2 disease profiles revealed three distinct branches, (Fig. 1C; Supplementary Fig. 1). Branch 1 (Fig. 1C, red) was the largest and contained a range of diseases primarily related to metabolism, vascular disorders, mood, and neurodegenerative disorders. Subgroups within this cluster recapitulate known associations between diseases. For example, diabetes-related disorders largely cluster together, as do vascular- and neurodegeneration-related disorders. Branch 2 (Fig. 1C, blue) was primarily composed of immune-system disorders including autoimmune diseases, multiple sclerosis, allergic reaction, and fever. Branch 3 (Fig. 1C, green) was primarily composed of neuropsychiatric disorders, including depression, addiction, bipolar, anxiety, and learning disorders. It also contained epilepsy, cardiovascular disorders, and pain, which are comorbid with psychiatric disorders [11–14]. Overall, these Cluster 2 branches show that shared mechanisms in branches are stronger than the original clusters, and the known mutual similarity of branch-revealed associations gives confidence to selectively investigate new connections between disorders and their underlying mechanisms.

### Cognition is the most affected biological process across all psychiatric cluster disorders

The high concentration of psychiatric disorders in Branch 3 (Fig. 1C, green, henceforth referred to as the “psychiatric cluster”) makes it the ideal place to investigate underlying mechanisms across psychiatric illnesses; thus, it remained the focus for subsequent analyses. This branch splits into four distinct subgroups (Fig. 1C, green, expansion).

**Fig. 2:**
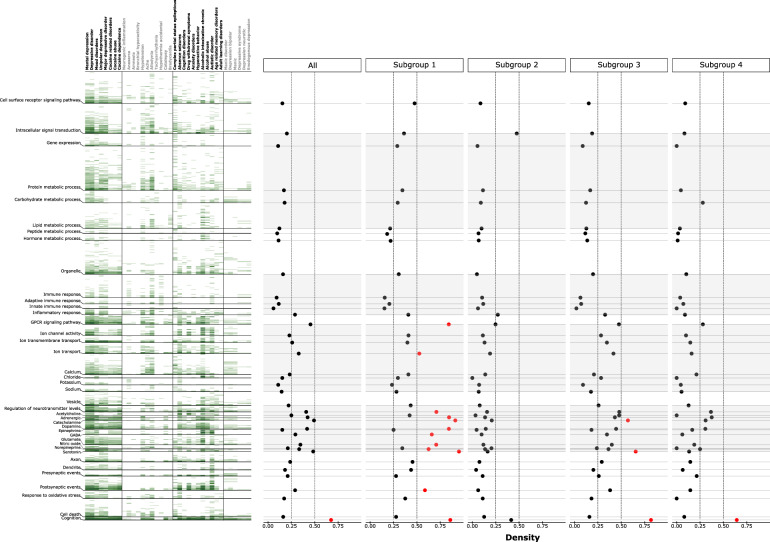
Pathway analysis of psychiatric cluster disorders. Left**:** Heatmap of significant (*q* < 0.05) and theme (left labels) filtered pathways associated with all psychiatric cluster disorders (top labels). The color-intensity (light to dark green) in the heatmap is proportional to −log10(*q* value). Right: Densities of each theme across all disorders and individual subgroups. The red dots represent the highest density themes crossing the threshold (arbitrary) of 0.5. Note the highest density of cognition across all disorders. See Supplementary Table 2 for further details.

GO analysis of gene-sets associated with psychiatric cluster revealed over 3000 GO pathways (*q* value < 0.05, Supplementary Table 2). To better understand the disease process, the pathways were organized into forty themes representing different levels of cellular and biological complexity (Fig. 2, left labels). To facilitate theme-centric quantitative comparison, we calculated a density index for each theme (Fig. 2, right). A density close to 1 indicates themes common across diseases, whereas a density close to 0 indicates themes unique to a few diseases. Across the entire psychiatric cluster, *cognition* shows the highest density (density ≈ 0.7). Other high-density (density ≈ 0.5) themes include *neurotransmission*, largely driven by *catecholamine* and *serotonin*, and signaling pathways, driven by *G-protein-coupled receptors*. Medium-density (density > 0.25) themes include other neurotransmitters, including *glutamate*, *GABA*, and *norepinephrine*; ion balance, driven by *transmembrane transport*; *postsynaptic events* and *inflammatory response*. Low-density (density < 0.25) themes include those related to the *immune system*, *metabolism*, *cell surface receptor signaling*, *intracellular signal transduction*, and *oxidative stress*.

While *cognition* is the most consistently affected process, comparing themes across subgroups reveals differences between the mechanisms underlying this phenotype. Subgroup 1, containing Major depressive disorder and cocaine addiction, exhibits the highest densities across all the subgroups for themes related to *adrenergic*, *catecholamine*, *dopamine*, *GABA*, *glutamate*, *norepinephrine*, and *serotonin*-related neurotransmission, possibly involving the slow *G-protein-coupled receptor signaling*, which too showed a high density. Interestingly, as compared to increased neurotransmission, Subgroup 1 showed reduced *presynaptic* events relative to *postsynaptic* events, suggesting a reduced input functionality.

Consistent with the known comorbidities of psychiatric illnesses with the autonomic response [13, 15], Subgroup 2, contained cardiovascular conditions and pain. All themes (including cognition) in this subgroup showed low density, suggesting subtle changes governed by unique mechanisms. Aside from cognition, the highest density themes in this subgroup are *G-protein-coupled receptor signaling*, *ion channel activity*, *inflammatory response*, and neurotransmission related to *nitric oxide* and *catecholamine*. As indicated by the low theme-density correlations (Supplementary Fig. 2), this subgroup differs most from the other subgroups, likely due to the peripheral origin (autonomic response) and greater relative contribution of immune system dysfunction than alterations to neurotransmission.

Subgroup 3, containing epilepsy, anxiety, alcohol abuse, and attention-deficit/hyperactive disorder, showed high theme-density correlation with Subgroup 1. Density associated with cognition was particularly similar in both subgroups (≈0.8). Besides *cognition*, the highest density themes in this subgroup are related to neurotransmission, largely driven by *serotonin* and *catecholamine*. Although lower in density, *inflammatory response*, *postsynaptic events*, and *ion balance* showed changes resembling Subgroup 1. Overall, the similarity between the two subgroups indicates that alcohol and cocaine abuse have similar downstream effects and mechanisms widely shared with depression, anxiety, and epilepsy. The difference separating the two appears to be in the relative severity of the affected processes.

Subgroup 4, containing bipolar disorder, mostly exhibits medium-density theme indices showing similar correlations across subgroups. Neurotransmission associated with *adrenergic*, *catecholamine*, and *dopamine* was most affected in this subgroup. Interestingly, the density of *carbohydrate metabolic process* is uniquely high in this group, suggesting that metabolic processes may play a larger role in bipolar disorder over other psychiatric disorders. Indeed, there are evidences for dysregulated metabolic processes in manic states [16, 17].

Overall, the unsupervised clustering of the highly comorbid psychiatric disorders suggests that neurotransmission, mostly associated with monoamines and governed by *G-couple protein receptors*, is the key shared process across psychiatric diseases that contributes to cognitive dysfunction. In contrast, pathways associated with *cell surface receptors*, *signal transductions*, and *metabolic process* are unique to a few disorders.

### Druggable mechanisms support the involvement of neurotransmission across psychiatric cluster disorders

Therapeutic or disease-inducing drugs with known mode of action (MOA) can expand our understanding of the underlying disease pathology [18]. By comparing the psychiatric disease-associated gene-sets with those of known drugs from the connectivity map [19], a database cataloging transcriptomic response of several cell lines against known drugs, we identified 132 relevant drugs, belonging to 64 different MOAs (Supplementary Table 3).

The most frequent MOAs across the entire psychiatric cluster involved *dopamine receptors* (15/64), *adrenergic receptors* (14/64), *glucocorticoid receptors* (8/64), and *ATPases activity* (6/64). Interestingly, these MOAs remained most frequent across each subgroup, considered individually (Supplementary Fig. 3). Within each subgroup, Subgroup 1 showed the most with 44 MOAs, whereas Subgroup 4 showed the least with 13 MOAs. Subgroup 3 and Subgroup 4 showed 20 and 24 different MOAs, respectively. Looking at specific drugs, those with the strongest density were *helveticoside* (targeting *ATPases*), *thioridazine* (targeting *dopamine receptors*), *clioquinol* (targeting *opioid receptors*), and *anisomycin* (targeting *DNA synthesis*). Overall, the drug-target analysis supports the pathway analysis findings that most psychiatric disorders feature dysregulated neurotransmission and the diverse MOAs points towards the heterogeneous nature of psychiatric disease origins [20].

### Interneurons are most affected cell-types across psychiatric cluster disorders

**Fig. 3:**
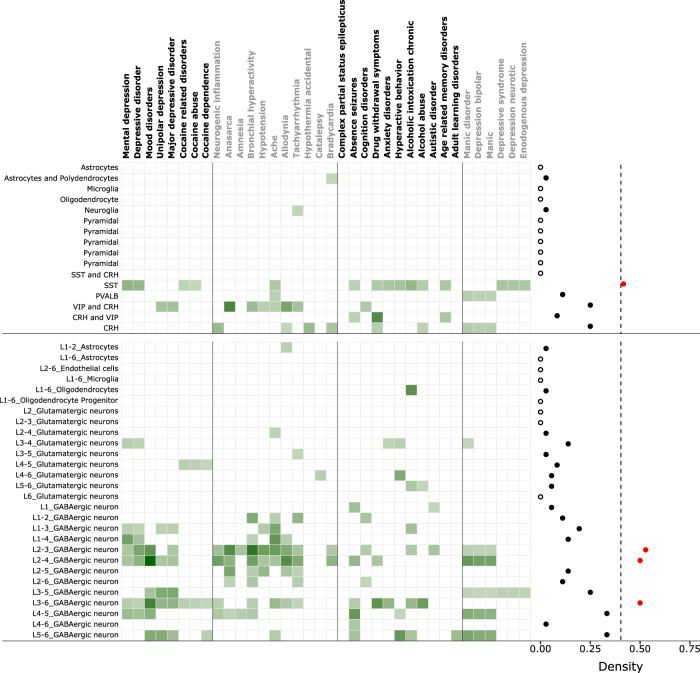
Cell-type analysis of psychiatric cluster disorders. Enrichment of different cell-types (top-left labels) and layer-specific cell-types (bottom-left labels) markers across psychiatric cluster disorders (top labels). Right: Density of each cell-type (top) and layer-specific cell-types (bottom) across all disorders. The filled red dots crossing the threshold (arbitrary) of 0.4, represent high-density cell-types whereas hollow dot represents a cell-type with zero density. Note the highest density of SST-positive interneurons and layer 2/3 specific interneurons across all disorders. The color-intensity (light to dark green) in the heatmap is proportional to −log10(*q* value).

Alterations in various layer-specific subtypes of neurons and glia is an important factor driving disease mechanisms and could differentially contribute to disease pathology [21, 22]. Using human-specific markers from two independent studies, without and with layer specificity, taken from anterior cingulate cortex [23] and middle temporal gyrus [24], respectively, we assessed the enrichment of neuronal and non-neuronal cell-types in the psychiatric cluster (Fig. 3). Based on cell-specific markers (Fig. 3, top), Subgroup 1 diseases were enriched in somatostatin (*SST*) and corticotrophin-releasing hormone (*CRH*) positive interneurons in a non-overlapping manner. Mental depression and cocaine addiction were enriched in *SST*-positive interneurons, whereas unipolar depression and major depression were enriched in *CRH*-positive interneurons. Subgroup 2 diseases were enriched with more diverse cell-types and consistent with their role in inflammatory diseases, were also enriched in neuroglia and oligodendrocytes. However, *CRH* and vasoactive intestinal polypeptide (*VIP*) co-expressing *CRH* neurons were most abundant in this cluster. Subgroup 3, consistent with the high theme-centric correlation with Subgroup 1, was also enriched in *SST* and *CRH*-positive interneurons. In Subgroup 4, endogenous, neurotic, and syndromic form of depression, similar to mental depression observed in Subgroup 1, were enriched in *SST* interneurons, whereas those with bipolar disorder were enriched in Parvalbumin (*PV*)-positive and *CRH*-positive interneurons.

Consistent with cell-specific markers, layer-specific markers also showed excessive enrichment in interneurons, mostly distributed across all layers (Fig. 3, bottom). However, based on cell-specific density index calculated across all the disorders in psychiatric cluster, GABAergic interneurons of layer 2/3 show the highest enrichment (density = 0.52). Associations with other cell-types were again weaker and less consistent. Within glutamatergic neurons, those in layer 3/4 showed the highest density of enrichment. Astrocytes and oligodendrocytes were involved with very few diseases with a density index of ~0.02. Overall, holding true for markers from two independent studies, interneurons were the most consistently implicated cell-type across all psychiatric subgroups and the most affected interneurons and glutamatergic neurons belong to superficial layer 2/3 and 3/4, respectively.

### Most chromosomes are associated with psychiatric cluster disorders

**Fig. 4:**
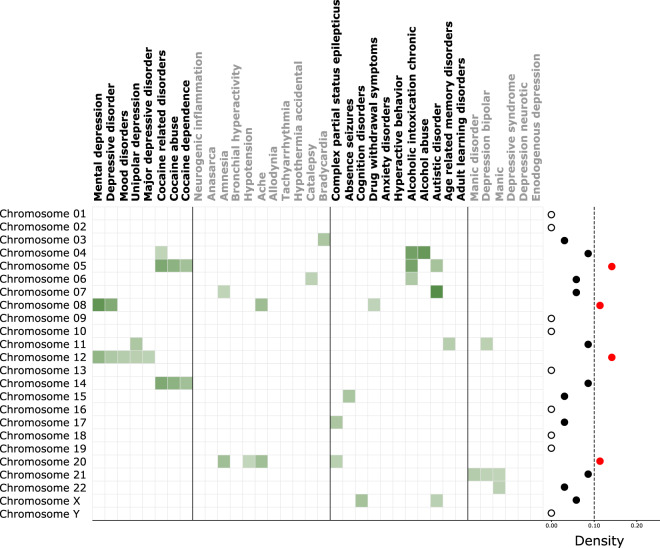
Chromosome overrepresentation analysis of psychiatric cluster disorders. Overrepresentation of different chromosomes across psychiatric cluster disorders (top labels). The filled red dots crossing the threshold (arbitrary) of 0.1, represent high-density chromosomes whereas hollow dots represent chromosomes with zero density.

Disease-associated gene-sets can be biased towards specific chromosomes, abnormalities in which can potentially explain the psychiatric disorders within each subgroup. Thus, we looked for chromosomal overrepresentation within the psychiatric subgroups (Fig. 4). 15/23 chromosomes showed overrepresentation across the psychiatric cluster, the densest of which were chromosome 5, 8, 12, and 20. Chromosome 5, was overrepresented in disorders associated with addiction (cocaine and alcohol) and autism. Chromosome 8 showed the most diversity and was associated with disorders across three subgroups. Chromosome 12, was overrepresented exclusively in Subgroup 1, representing mood and depressive disorders. Chromosome 20 was overrepresented in Subgroup 2 and complex partial status epilepticus in Subgroup 3.

Within each subgroup, Subgroup 1 showed association with five chromosomes overrepresented across all 8/8 disorders observed here. Interestingly, a split developed, with chromosomes 8 and 12 being more related to depression and chromosomes 5 and 14 being more related to cocaine addiction. Subgroup 2 showed association with five chromosomes across 5/11 diseases observed here, with chromosome 20 being most affected. Subgroup 3 was the most diverse, being associated with nine chromosomes across 8/11 disorders observed here. Autism and cognitive disorder, belonging to this subgroup, were the only disorders overrepresented in X-chromosomes. Subgroup 4 was the least diverse with only three chromosomes overrepresented across 3/6 disorders observed here. Chromosome 21 showed the highest density in this subgroup associated with bipolar and related disorders.

### Chromosomal overrepresentation drives the similarity between psychiatric cluster disorders

Next, we reasoned that, of the four levels of biological complexity (pathways, cell-type, drug-target, and chromosomes), the one which drives the similarity between disorders, should cluster them in agreement with the psychiatric cluster (Fig. 1C, green; Supplementary Fig. 4). Based on RI, a measure of similarity between two clusters [25], we observed moderate but significant similarity between psychiatric cluster and chromosome overrepresentation based clustering of disorders (RI = 0.70, *p* value < 3 × 10^−03^). A weak trend level similarity was also observed between psychiatric cluster and cell-enrichment based clustering (RI = 0.65, *p* value < 0.07). Finally, among different levels, consistent with the above results, moderated but significant similarity was observed between pathway- and MOA-based clustering (RI = 0.70, *p* value < 1 × 10^−04^). Thus, the similarity between disorders, to some extent, can be explained based on chromosomal instability and cellular correlates of the disorders.

## Discussion

The search for common molecular mechanisms across psychiatric disorders is an important future direction for the field. Here, we show that disease-specific gene-sets are not only sufficient to segregate diseases based on their intrinsic nature and point to similarities between comorbid conditions, but also to move towards a more mechanistic understanding of neural connectivity and disease origins.

**Fig. 5:**
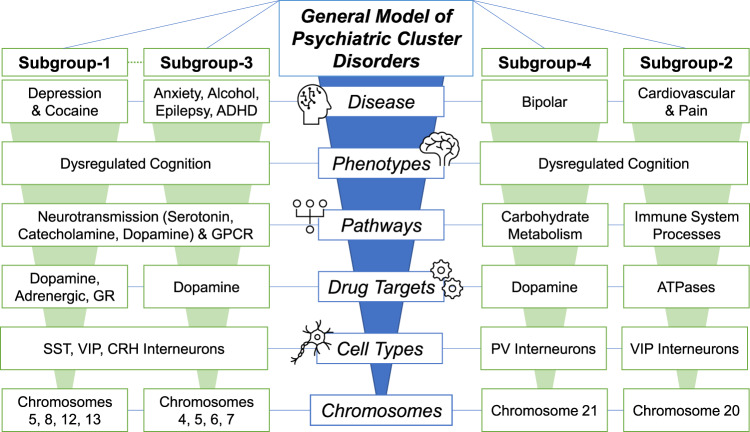
General model of psychiatric cluster disorders. Overall, the present analyses suggest that psychiatric disorders are impacted at physiologic levels of biological complexities, with changes at the chromosome level emanating up to cell-type, drug-target, and pathway levels. Collectively these alterations generate various phenotypes, largely related to cognition, which contribute to disease. The present subgroups show similarities and differences across all of these levels, with Subgroup 1 and Subgroup 3 showing the most similarities, and Subgroup 2 showing the most differences.

Starting with 763 disease-associated gene-sets, we uncovered three distinct disease clusters, demonstrating fundamental differences between psychiatric/metabolic-type disorders and cancer. Focusing on a cluster enriched in psychiatric diseases, we compared implicated pathways, drug-targets, cell-types and chromosomes. The most commonly dysregulated processes across all disorders involved neurotransmission, neuromodulation, and synaptic signaling. Whereas the most uniquely dysregulated processes across few disorders involved *immune system response*, signal-response coupling involving *cell surface-based signaling* and *intracellular signal transduction* and downstream response involving *gene expression* and *metabolic process*. Independently, the ubiquitous role of neurotransmission was also observed in drug-target based analysis. Furthermore, we showed that the similarity between psychiatric disorders is significantly driven by alterations at the chromosome, and to a lesser extent by the cell-types. This suggests that similarity between disorders disperses in a bottom-up fashion (Fig. 5) i.e., following subcellular chromosomal abnormality, the similarity persists at cellular level where it is affected most by neurotransmission and modulation, and disperses at pathway levels possibly through different outside stimulus as suggested by low density of signal-response coupling. Notably, the observed chromosomal overrepresentation was consistent with large bodies of literature on abnormalities of chromosome 5 in substance abuse [26, 27], chromosome 8 and 12 in lifetime major depression [28, 29] and anxiety-disorder [30] and chromosome 20 in epilepsy [31, 32]. Further support for similarity at chromosome level comes from a genome wide linkage study suggesting shared effects on five major psychiatric disorders [4], four of which are also reported in this study.

The gene-set based approach used here has several advantages over previous work using whole transcriptome approaches [2, 3], observing (only) similarities between a few disorders. First, unbiased clustering at a global level (Fig. 1B) uncovers shared biological origin of several disorders. For instance, with exception of diseases populating the overlapping boundaries of the three clusters, Cluster 1, populated by diseases of familial, sex-linked and autosomal origin, may broadly represent mendelian disorders and patterns that reflect inheritance; Cluster 2, populated by psychiatric disorders and comorbid conditions, may represent complex disorders; and Cluster 3, exclusively populated by cancer, may represent disorders of environmental origin. In this regard, assignment of schizophrenia, a major psychiatric disorder, to Cluster 1 is interesting. On one hand, it recognizes schizophrenia as a disorder with an inheritance element. On the other, it highlights the subtle differences between schizophrenia and psychiatric cluster disorders, possibly reflecting its high heterogeneity with known variations associated with developmental process [33], psychosocial stress [34], personality trait [35], time-point of onset [36], gender differences [37, 38], emotional experiences [39], and various behavioral component constructs [40], among others. While one would expect schizophrenia to fall in the psychiatric cluster, the fact that it did not is very intriguing and further supports the ability of this unbiased approach to lend novel insights into the field.

Second, this approach allows for exploration of a disease and its comorbidities independently at multiple biological levels. For instance, the comorbidity of cardiovascular disorder and pain (Subgroup 2, Fig. 1C) with psychiatric illnesses is well-studied in clinical and epidemiological set-up [13, 15, 41, 42] but not in postmortem molecular studies, where (1) such comorbidity data are usually not available or (2) the high collinearity between the comorbid conditions and disease state makes it difficult to segregate the two conditions for independent assessment. The present study circumvents these issues and supports the existence of such comorbidities from independent gene-sets. Note that being peripherally derived (autonomic response), the independent contribution of Subgroup 2 towards cognition is less; however, its presence within the psychiatric cluster suggests that a reduced set of cellular/biological process, involving *G-protein-coupled receptor signaling and catecholamines* neurotransmission (Fig. 2), may be common between autonomic alterations and psychiatric disorders.

### A general model of psychiatric cluster disorders

Cognition was the most affected process across all psychiatric cluster disorders, and its known pathophysiological association with neurotransmission can be seen at both the pathway (Fig. 2) and drug-target levels (Supplementary Fig. 3). Within different neurotransmitters, although at different densities, all subgroups showed the highest density of catecholamines. Among other neurotransmitters, serotonin-related pathways were densest in the highly correlated Subgroups 1 and 3, representing mood and addiction, respectively, but not in Subgroups 2 and 4, representing neuroinflammation and bipolar disorder, respectively. Overall suggesting that, while neurotransmission is a common mechanism between these disorders, the differences may emerge due to dissimilar neurotransmitter systems.

This raises the question on how things are governed at the cell-type level, which as suggested by our results, also drives similarity between the disorders to some extent. Notably, at the cell-type level almost all disorders were enriched with dendrite-targeting *SST* and *VIP* interneurons suggesting that these disorders are largely related to context (all inputs except the one of interest) dependent integration of information input to pyramidal neurons, a function largely associated with these interneurons [43]. *VIP* interneurons, by disinhibiting the *SST* interneurons, also influence the impact of most noxious or negative information input [44]. In this regard, their highest enrichment in Subgroup 2 suggests their influence on ache, allodynia, and tachyarrhythmia, which are all comorbid disorders involving a noxious stimulus of pain. Finally, consistent with the role of corticotropin-releasing hormone in stress, we also observed the enrichment of *CRH*-positive interneurons which are mostly co-expressed with *SST* or *VIP* interneurons [23]. One notable exception here is the enrichment of *PV*-positive interneurons in ache and bipolar-disorders. Note that *PV*-interneurons, unlike *SST* and *VIP* interneurons, largely target axon-initial segments and govern adaptation of output from pyramidal neurons [45]. As such, their enrichment in bipolar-disorders aligns with its previously observed similarity with schizophrenia, a disorder associated with abnormal output [2, 3].

The information input coming to these cell-types are potentially long-ranged, as suggested by higher enrichment of distantly produced monoamines, or short-ranged, as suggested by overall enrichment of local glutamatergic-transmission. Further support for long-distance input comes from the enriched *VIP* interneurons which are influenced by long-distance serotonergic and cholinergic afferents [44]. Finally, enrichment of neuronal markers across all layers also suggests broad-spectrum behavioral, cognitive, and autonomic input from different brain areas [46]. However, layer 3/4, receiving thalamocortical input [47], showed high density for excitatory neurons; whereas layer 2/3, responsible for inhibiting layer-1 re-entrant connections [48] from adjacent areas, showed high density for *SST* interneurons.

Overall, the cell and layer-specific enrichment suggests that disorders in the studied psychiatric cluster disorders are influenced by lack of adaptation to input coming from diverse contexts. Given the common presence of this phenotype, this inflexibility represents an intriguing process at the root of psychiatric disease that should be the focus of future mechanistic and therapeutic studies.

### Limitations and future direction

This top-down analysis was conducted using unbiased disease-associated gene-sets. However, the gene-sets used do not include direction (upregulation or downregulation). The conclusions we drew for cellular-associates are under the assumptions that the gene-sets used in the study are universal signatures of these disorders regardless of brain region. As such, future mechanistic studies should consider these variables. Future studies should also incorporate other central-nervous-system disorders to generate a broad computational framework of microcircuit dysfunction. A novel signature-based classification of psychiatric disorders based upon such a framework would be a valuable extension of these results and may point to more precise, therapeutically relevant mechanisms.

## Supplementary information

Supplementary Information

Supplementary Figure 1

Supplementary Figure 2

Supplementary Figure 3

Supplementary Figure 4

Supplementary Table 1

Supplementary Table 2

Supplementary Table 3
