# Supplementary material for: Similarities and dissimilarities between psychiatric cluster disorders
Source: Mol Psychiatry. 2021 Jan 27;26(9):4853–63. doi: 10.1038/s41380-021-01030-3 (PMC8313609; doi:10.1038/s41380-021-01030-3)
Supplement: Supplementary file 1 — Supplementary Information [file 41380_2021_1030_MOESM1_ESM.pdf]

-SUPPLEMENTARY INFORMATION FOR-

Similarities and Dissimilarities Between Psychiatric Cluster Disorders

Marissa A. Smail, BS[1,2], Xiaojun Wu, PhD[3], Nicholas D. Henkel, BA[3], Hunter M. Eby, BS[3],

James P. Herman, PhD[1,4,5], Robert E. McCullumsmith, MD, PhD[3,6], Rammohan Shukla, PhD[3*]

Table of Contents

SUPPLEMENTARY METHODS 2

Disease-Disease similarity 2

Filtering for psychiatric-cluster 2

Gene-Ontology analysis 2

Density-Index 3

Cell-Type and Drug-Target Enrichment Analysis 3

Chromosome overrepresentation-analysis 4

Rand-index 4

SUPPLEMENTARY FIGURES (each as separate PDF file) 5

Supplementary Figure 1: Cluster-2 hierarchical dendrogram labeled 5

Supplementary Figure 2: Theme-density correlations between psychiatric cluster subgroups 5

Supplementary Figure 3: Drug-target frequency across psychiatric cluster 5

Supplementary Figure 4: Comparing hierarchical dendrogram for Rand-index based similarity 5

SUPPLEMENTARY TABLES (each as separate excel file) 6

Supplementary Table 1: Jaccard matrix for all disease-associated gene sets 6

Supplementary Table 2: Detailed results of pathway analysis 6

Supplementary Table 3: Detailed results of drug target analysis 6

REFERENCES 7

SUPPLEMENTARY METHODS:

Disease-Disease similarity: Curated disease-associated gene-sets were downloaded from DisGeNET. To avoid size-related bias and improve the specificity of pathway profiles (1–3), we restricted our analysis to 763 diseases with gene-set size between 10 to 500 (Supplementary Table 1). Several diseases showed similar annotations. However, as minor differences in related gene-sets could have significant impacts on the resulting molecular mechanisms, any disease-associated gene-set, even with one gene difference from other gene-sets, was considered in the analysis. Such similarity also reflects biological parsimony and can serve to internally validate our analysis. Pairwise-similarity between disease-associated gene-sets was calculated using Jaccard similarity-index (4) (gene-overlap package in R; R version 3.6.0).

Filtering for psychiatric-cluster: To narrow our focus on a more comprehensive examination of shared and unique processes in psychiatric disorders, we took a three-step top-down approach to filtering an unbiased cluster of psychiatric disorders (Figure 1A). First, using principal component analysis (PCA) of Jaccard similarity-matrix, global clusters of diseases were identified (Figure 1B). Initial examination of global clusters revealed clusters of highly similar diseases. Second, using the original Jaccard similarity-matrix filtered for diseases in a global cluster enriched with psychiatric disorders, a Euclidean distance-based hierarchical cluster was generated using a minimum variance-based clustering (Figure 1C) performed using the hclust package in R-3.6.0. Finally, using the cuttree function, a dendrogram cluster of 36 disorders (Figure 1C, green expansion), most enriched with psychiatric disorders and associated comorbidities (referred to as the “psychiatric-cluster”) was extracted and used for all further analyses.

Gene-Ontology analysis: Pathways affected in different diseases were determined using hypergeometric overlap analysis implemented by gene-overlap package in R-3.6.0. Given two gene-lists, a significance test for their overlap can be described using hypergeometric distribution performed with a genomic background representing universe of known genes (21,196 genes,

default used by the package). The null-hypothesis represents an odd-ratio <1 whereas the alternate hypothesis represents an odd-ratio >1. The significant overlap (q-value<0.05) of disease-associated gene-sets were tested against gene-ontology (GO) pathways associated with Biological-Process (GOBP), Molecular-Function (GOMF), and Cellular-Component (GOCC). Updated list of GO-pathways was obtained from the Bader-lab (http://download.baderlab.org/EM_Genesets/). To compare the effect of pathways across different diseases, the -log10(q-value) was used to generate the heatmap (Figure 2). To better identify the character of biological changes, in the overlap results, a focused analysis of forty a-priori functional themes was performed. As describe in our previous study (5), the pathways were filtered based on the parent-child association between GO-terms in our list of significant pathways (child-pathways) and hand-picked parent-pathways representing the a-priori theme from the GO-database.

Density-Index: To quantitatively summarize how common (close to 1) or unique (close to 0) a theme is across different subgroups of psychiatric-disorders; we devised a density-index. For a given ***r*** **x** ***c*** matrix of -log10(q-value), a density-index is obtained as:

$$Density = 1 - \left(\frac{count\ of\ \ zero\ elements\ in\ the\ matrix}{r\ x\ c}\right)$$

Where ***r*** and ***c*** represent the number of pathways in a theme and number of disorders in a psychiatric subgroup, respectively. A zero element represents a non-significant disease-pathway relationship. For density associated with individual pathways, cell-types, drugs and chromosomes a vector representing the number of disorders (=36) in the psychiatric-cluster was used. In such an instance, the numerator of the above density formula represents count of zero elements in a vector. Whereas ***r*** and ***c*** in the denominator are constant holding a fixed value of 1 and 36, respectively.

Cell-Type and Drug-Target Enrichment Analysis: Enrichment of layer independent and layer specific neuronal cell-types and drug induced molecular signatures in the psychiatric cluster

associated gene-sets was calculated using hypergeometric overlap analysis described above. Human layer independent cell-type markers where used from Shukla *et. al.* [15] and the layer specific markers were used from Hodge *et. al.* [16]. For layer specific markers, only broad categories of cell types (GABAergic, glutamatergic, astrocytes, microglia and oligodendrocytes) stratified by different layers of middle temporal gyrus were used. Drug specific gene-markers were downloaded from the Enricher library of gene-sets (8). In order to understand the druggable-mechanism and targets involved, gene-markers of drugs with known modes of action (MOA) and targets were used.

Chromosome overrepresentation-analysis: To access the chromosomal enrichment of each gene-set in the psychiatry cluster we used Fisher's exact test. A non-redundant list of genes within each chromosome was downloaded from Hugo Gene Nomenclature Committee (9) and used as background.

Rand-index: To compare the clustering of disorders in psychiatric-cluster with their clustering outcome using pathway, cell-type, drug-target, and chromosome enrichment profiles, we used Rand-index (fossil-package in R). The index, ranges between 0 and 1, where 1 represents identical clustering outcome. The ordered vector of cluster labels generated by each clustering outcome were used as input for the comparison. Significance was generated by two-tailed randomization-test using 1000 resampling permutations of psychiatric-cluster labels as reference. (see supplementary figure 4 for additional details).

SUPPLEMENTARY FIGURES (<u>each as separate PDF file</u>)

Supplementary Figure 1: Cluster-2 hierarchical dendrogram labeled. Disorders corresponding to the red, blue, and green branches of the Cluster-2 dendrogram presented in Figure 1B.

Supplementary Figure 2: Theme-density correlations between psychiatric cluster subgroups. Correlation coefficient (r) are presented above the diagonal and correlation plots are presented below the diagonal. Each dot represents the theme-density.

Supplementary Figure 3: Drug-target frequency across psychiatric cluster. Proportional representation of different drug-target in the psychiatric cluster as a whole and within each subgroup. Only top drug-targets are shown.

Supplementary Figure 4: Comparing hierarchical dendrogram for Rand-index based similarity. Top: comparison of psychiatry cluster dendrogram (corresponds to figure 1B, green) with chromosome and cell-type based dendrogram. **Bottom:** comparison of pathway-based dendrogram with drug-target based dendrogram. Each dashed line (red) shows the dendrogram-cut and corresponding subgroups (black and gray text) of disease labels on the right. In order to generate significance (see methods), for each row (top and bottom) the cluster labels of dendrogram on the left of dashed line (black) was used as reference for resampling based premutation test. Note, only significant comparisons are shown.

SUPPLEMENTARY TABLES (each as separate excel file)

Supplementary Table 1: Jaccard matrix for all disease-associated gene sets and corresponding assignments to Cluster-1, Cluster-2, and Cluster-3 (corresponds to Figure 1A)

- **Worksheet-1 [JaccardMatrix (JM)]:** Jaccard matrix for all disease-associated gene sets. Similarity measurements for initial 763 disease-associated gene sets. Values correspond to percent similarity between gene-sets
- **Worksheet-2 [DiseasePerCluster]:** List of all disease in each cluster, as determined by principal component analysis (corresponds to Figure 1A)
- **Worksheet-3 [JM_Cluster1]:** Jaccard matrix for Cluster-1
- **Worksheet-4 [JM_Cluster2]:** Jaccard matrix for Cluster-2
- **Worksheet-5 [JM_Cluster3]:** Jaccard matrix for Cluster-3

Supplementary Table 2: Detailed results of pathway analysis (corresponds to Figure 2).

- **Worksheet-1 [All_Pathways]:** All unfiltered pathways (row) and corresponding -log10(q-values) per disorder (column) is provided. Column "AM" shows the density of each pathway across all disorders and column "AN" shows the disorder with highest -log10(q-values) value.
- **Worksheet-2 [Filtered_Pathways]:** Pathway filtered for corresponding themes (column "AM" and left labels in Figure 2).
- **Worksheet-3 [ThemeDensity_Index]**: Theme density across all disorders and individual subgroups. The correlation plot matrix for each theme density is shown in Supplementary Figure 2.

Supplementary Table 3: Detailed results of drug target analysis

- **Worksheet-1 [Drugs-MOA]:** Description (Molecule, MOA, MOA slim, CMap class and target) of all the 132 significantly enriched drugs downloaded from connectivity map data base. Note that MOA slim annotations do not have directions (agonist/antagonist or inhibitor/enhancer) associated with it. As the gene-sets used in the present study do not have directions (up- or down-regulated), we used only MOA slim to describe all the drugs in this study
- **Worksheet-2 [Drugs-DiseaseMOA_all]:** Drug (rows) and corresponding -log10(q-values) per disorder (column) is provided. A summary of the frequency of each drug target is provided in columns AJ and AK.
- **Worksheet-3 [Drugs-DiseaseMOA1]:** Drug-target analysis for Subgroup-1
- **Worksheet-4 [Drugs-DiseaseMOA2]:** Drug-target analysis for Subgroup-2
- **Worksheet-5 [Drugs-DiseaseMOA3]:** Drug-target analysis for Subgroup-3
- **Worksheet-6 [Drugs-DiseaseMOA4]:** Drug-target analysis for Subgroup-4

REFERENCES:

1. Williams AG, Thomas S, Wyman SK, et al.: RNA-seq Data: Challenges in and Recommendations for Experimental Design and Analysis. Curr Protoc Hum Genet 2014;
2. Holmans P: Statistical Methods for Pathway Analysis of Genome-Wide Data for Association with Complex Genetic Traits. 2010
3. Wang K, Li M, Hakonarson H: Analysing biological pathways in genome-wide association studies. Nat Rev Genet 2010;
4. Bass JIF, Diallo A, Nelson J, et al.: Using networks to measure similarity between genes: Association index selection. Nat Methods 2013;
5. Shukla R, Prevot TD, French L, et al.: The relative contributions of cell-dependent cortical microcircuit aging to cognition and anxiety [Internet]. Biol Psychiatry 2018; [cited 2018 Oct 9] Available from: https://www.sciencedirect.com/science/article/pii/S0006322318318900?via%3Dihub
6. Shukla R, Sibille E, Newton D, et al.: Molecular Characterization of Depression Trait and State [Internet]. bioRxiv 2020; 2020.04.24.058610[cited 2020 Apr 28] Available from: https://www.biorxiv.org/content/10.1101/2020.04.24.058610v1
7. Hodge RD, Bakken TE, Miller JA, et al.: Conserved cell types with divergent features in human versus mouse cortex. Nature 2019;
8. Kuleshov M V., Jones MR, Rouillard AD, et al.: Enrichr: a comprehensive gene set enrichment analysis web server 2016 update. Nucleic Acids Res 2016;
9. Yates B, Braschi B, Gray KA, et al.: Genenames.org: The HGNC and VGNC resources in 2017. Nucleic Acids Res 2017;
