# Supplementary figures and images for: Similarities and dissimilarities between psychiatric cluster disorders
Source: Mol Psychiatry. 2021 Jan 27;26(9):4853–63. doi: 10.1038/s41380-021-01030-3 (PMC8313609; doi:10.1038/s41380-021-01030-3)

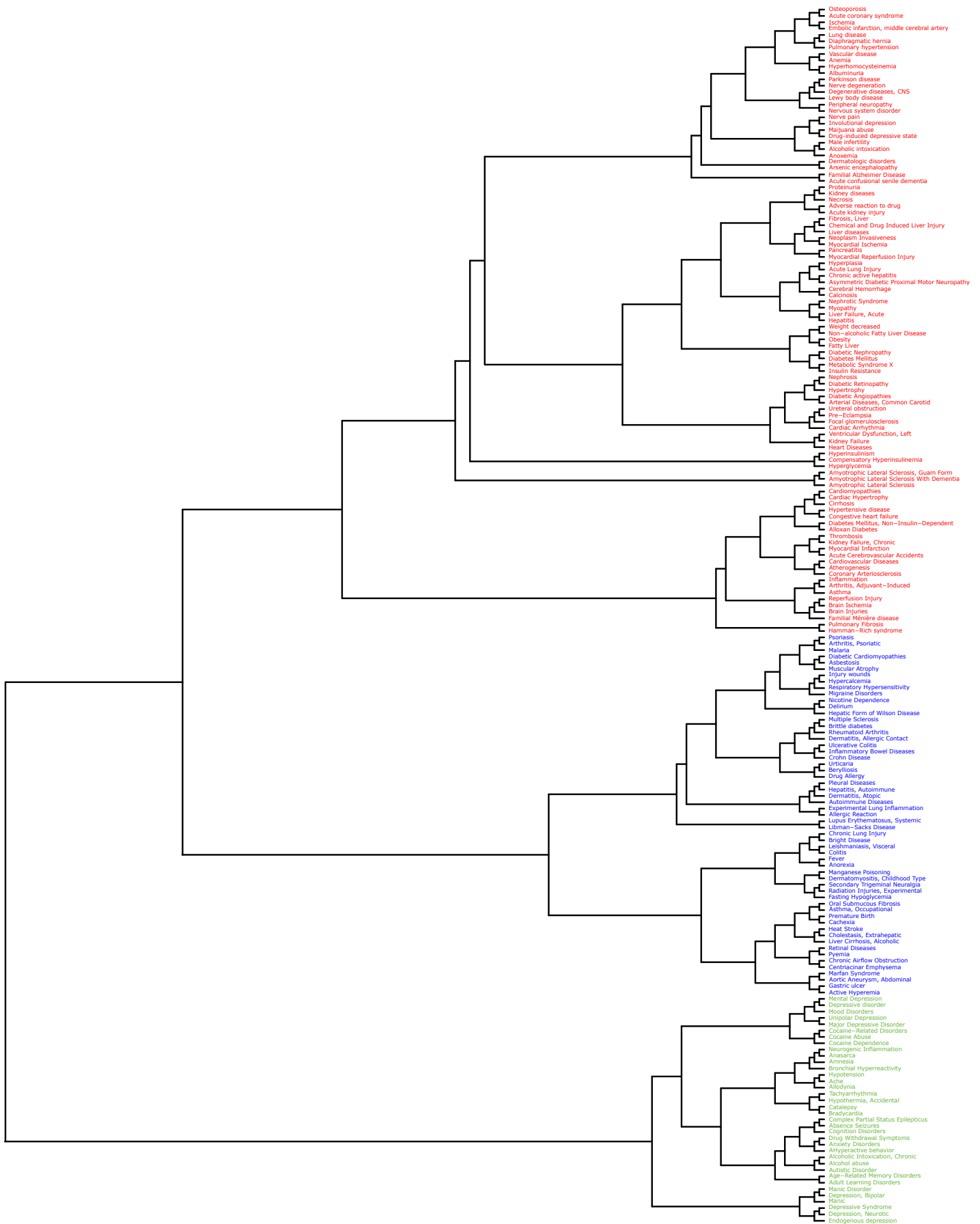

Supplementary Figure 1

Supplement: Supplementary file 2 — Supplementary Figure 1 [file 41380_2021_1030_MOESM2_ESM.pdf]

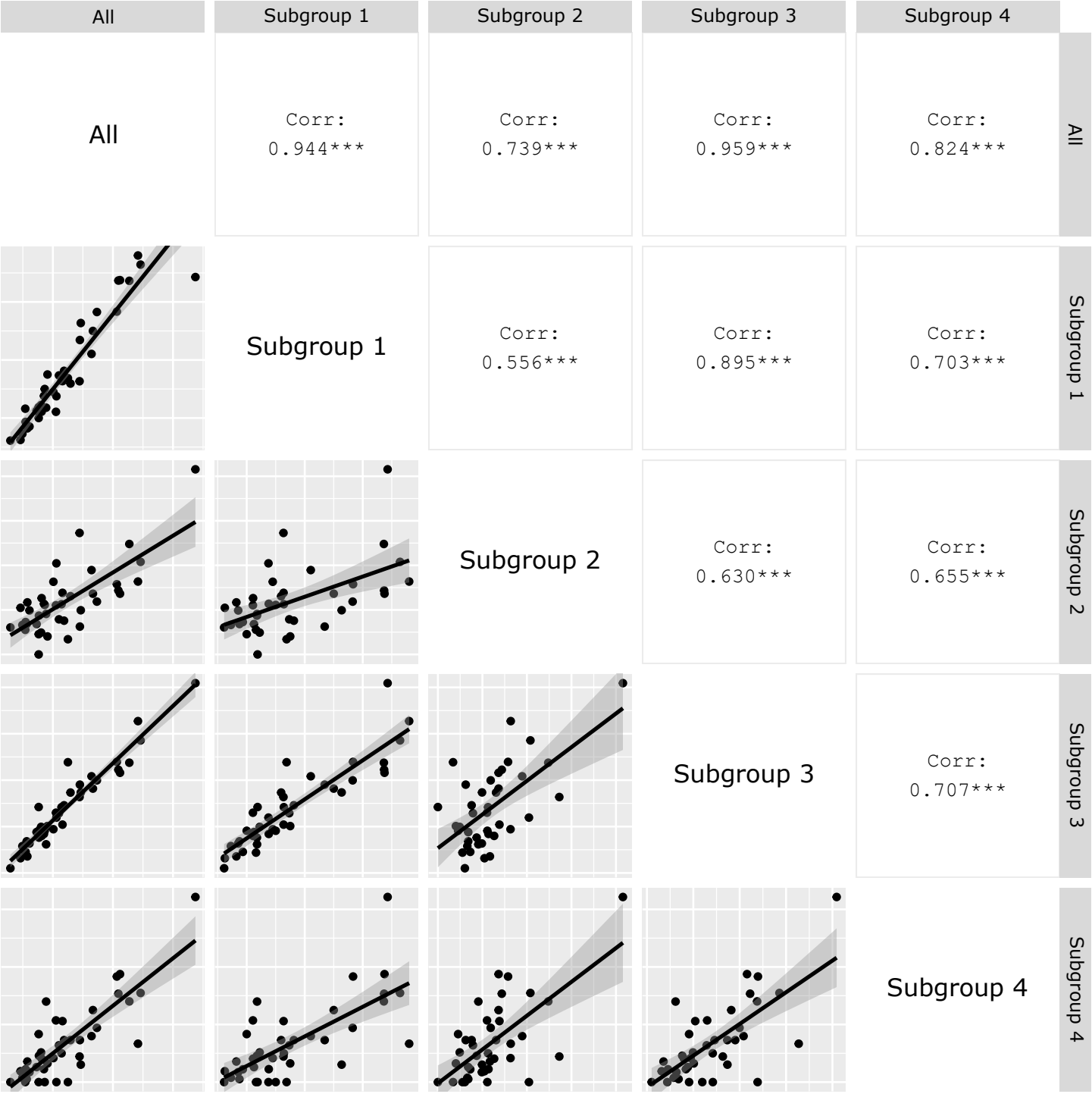

Supplementary Figure 2

Supplement: Supplementary file 3 — Supplementary Figure 2 [file 41380_2021_1030_MOESM3_ESM.pdf]

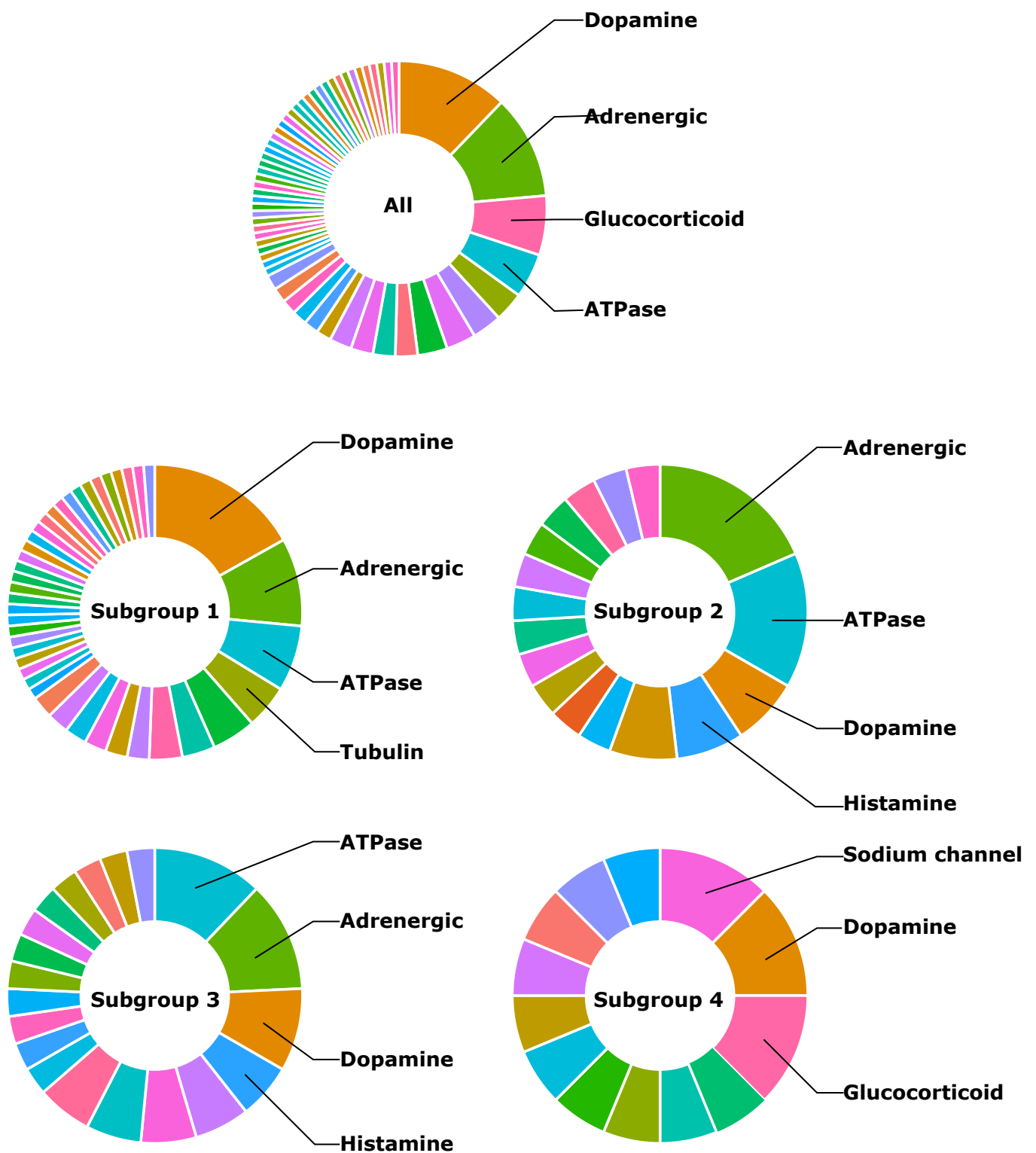

Supplementary Figure 3

Supplement: Supplementary file 4 — Supplementary Figure 3 [file 41380_2021_1030_MOESM4_ESM.pdf]
