# Supplementary material for: Similarities and dissimilarities between psychiatric cluster disorders
Source: Mol Psychiatry. 2021 Jan 27;26(9):4853–63. doi: 10.1038/s41380-021-01030-3 (PMC8313609; doi:10.1038/s41380-021-01030-3)
Supplement: Supplementary file 5 — Supplementary Figure 4 [file 41380_2021_1030_MOESM5_ESM.pdf]

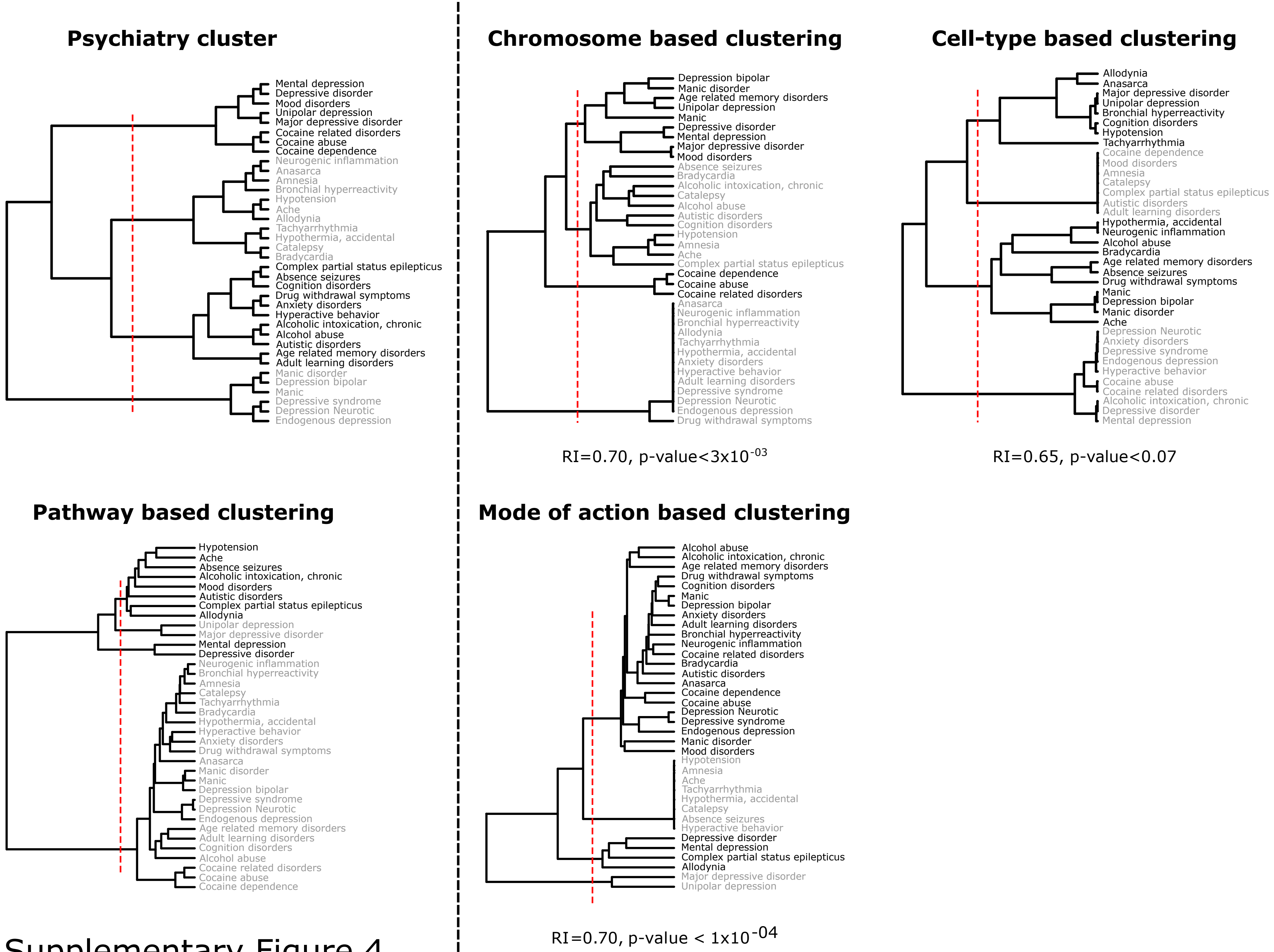

Supplementary Figure 4
